# Diverse histone modifications on histone 3 lysine 9 and their relation to DNA methylation in specifying gene silencing

**DOI:** 10.1186/1471-2164-8-131

**Published:** 2007-05-24

**Authors:** Jiejun Wu, Shu-Huei Wang, Dustin Potter, Joseph C Liu, Laura T Smith, Yue-Zhong Wu, Tim H-M Huang, Christoph Plass

**Affiliations:** 1 Department of Molecular Genetics, The Ohio State University, Columbus, OH, USA; 2 Department of Molecular Virology, Immunology, and Medical Genetics, Division of Human Cancer Genetics, The Comprehensive Cancer Center, The Ohio State University, Columbus, OH, USA

## Abstract

**Background:**

Previous studies of individual genes have shown that in a self-enforcing way, dimethylation at histone 3 lysine 9 (dimethyl-H3K9) and DNA methylation cooperate to maintain a repressive mode of inactive genes. Less clear is whether this cooperation is generalized in mammalian genomes, such as mouse genome. Here we use epigenomic tools to simultaneously interrogate chromatin modifications and DNA methylation in a mouse leukemia cell line, L1210.

**Results:**

Histone modifications on H3K9 and DNA methylation in L1210 were profiled by both global CpG island array and custom mouse promoter array analysis. We used chromatin immunoprecipitation microarray (ChIP-chip) to examine acetyl-H3K9 and dimethyl-H3K9. We found that the relative level of acetyl-H3K9 at different chromatin positions has a wider range of distribution than that of dimethyl-H3K9. We then used differential methylation hybridization (DMH) and the restriction landmark genome scanning (RLGS) to analyze the DNA methylation status of the same targets investigated by ChIP-chip. The results of epigenomic profiling, which have been independently confirmed for individual loci, show an inverse relationship between DNA methylation and histone acetylation in regulating gene silencing. In contrast to the previous notion, dimethyl-H3K9 seems to be less distinct in specifying silencing for the genes tested.

**Conclusion:**

This study demonstrates in L1210 leukemia cells a diverse relationship between histone modifications and DNA methylation in the maintenance of gene silencing. Acetyl-H3K9 shows an inverse relationship between DNA methylation and histone acetylation in regulating gene silencing as expected. However, dimethyl-H3K9 seems to be less distinct in relation to promoter methylation. Meanwhile, a combination of epigenomic tools is of help in understanding the heterogeneity of epigenetic regulation, which may further our vision accumulated from single-gene studies.

## Background

It is well known that DNA methylation plays a repressive role in gene transcription, both in heterochromatin and in repressed, protein-coding, euchromatin [1]. Recent work demonstrated that DNA methylation cooperates with histone modifications to perform this repressive function [2]. Acetylation and methylation on histone 3 lysine 9 (acetyl-H3K9 and methyl-H3K9, respectively) are two of the best studied modifications. Acetyl-H3K9 is known to be associated with active transcription, and methyl-H3K9 with repressed transcription [3]. To better understand the mechanisms of epigenetic regulation, it is necessary to clarify the crosstalk, including the distribution patterns, between these epigenetic markers [3]. Some reports showed the physical interaction between histone deacetylase and histone methyltransferase [4,5]. Meanwhile, removal of acetylation has been shown to be a necessary step for histone methyltransferase activity [4,5]. It is believed that histone acetylation and histone methylation act in concert to regulate gene transcription. Studies in fungi and plant, and, to a lesser degree, in mammals indicate that methyl-H3K9 may control DNA methylation in heterochromatin [6-8]. Current knowledge also supports the idea that repressive complexes, containing both methyl binding domain (MBD) proteins and histone deacetylases (HDACs), in combination with other repressor proteins, direct DNA methylation and subsequently transcriptional repression [9]. Additional evidence shows that DNA methylation impacts histone methylation and that DNA methylation might exert a positive feedback on lysine methylation [10-14]. To reconcile these two seemingly distinct mechanisms, a self-enforcing network of epigenetic regulation has been proposed: histone methylation impacts DNA methylation and histone acetylation which in turn impacts histone methylation [3,9].

The current self-enforcing model implies close correlation of histone modifications and DNA methylation, especially the crosstalk between methyl-H3K9 and DNA methylation [3]. However, recent reports demonstrated that these epigenetic markers have varying degrees of autonomy [10,15-22]. For example in *Arabidopsis*, Trichostatin A (TSA), a histone deacetylase inhibitor, and 5'-aza-2-deoxycytidine (AzadC), a demethylating agent, do not always produce redundant outcomes. Most surprisingly, they may even demonstrate antagonistic effects as opposed to the expected synergistic effects [16]. In *Arabidopsis*, where DNA methylation is not crucial for survival, methyl-H3K9 marks heterochromatin independent of DNA methylation [10]. In mammals, no close association between methyl-H3K9 and DNA methylation was discovered for: imprinted gene loci on distal chromosome 7 [17,18], the inactive X chromosome in ICF and Rett syndrome cells [19], *FMR1 *in fragile X syndrome [22], or *MGMT, LHR *in human cancers [20,21].

Here we analyze profiles of DNA methylation and histone modifications in a mouse leukemia genome to better understand the relationship between these epigenetic events. Using genome-wide data, we demonstrate that histone acetylation and histone methylation show a distinct degree of autonomy with respect to promoter methylation.

## Results

### Global profiling of acety-H3K9, dimethyl-H3K9, and DNA methylation in L1210 cells

**Figure 1:**
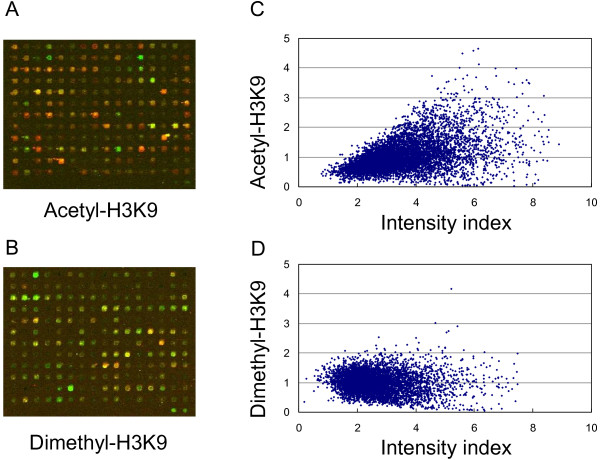
**The distribution of histone modifications screened by ChIP-chip with mouse CpG island array**. **A **and **B**. Representative results from the mouse CpG island arrays hybridized by ChIP DNA, labeled with Cy5, and input control, with Cy3. **C and D**. Scatter plot of histone modification level to density index. Histone modification level is indicated by the fold enrichment of ChIP DNA vs input DNA.

We first performed chromatin ChIP-chip on the mouse leukemia cell line, L1210, with antibodies detecting either acetyl-H3K9 or dimethyl-H3K9. ChIP products were hybridized onto the mouse 9.2K CpG island microarray. Immunoprecipitated DNAs from acetyl- or dimethyl-H3K9 ChIPs were compared individually with total genomic DNA input. Increased hybridization signals indicated an enrichment of a specific histone modification for a given CpG island locus (red signals in Figure 1A and 1B). The scatter plot, with fold changes plotted against geometric mean of signal intensities, showed that the relative level of acetyl-H3K9 has a wider range of distribution than the intensity index seen for dimethyl-H3K9 (Figure 1C and 1D).

**Figure 2:**
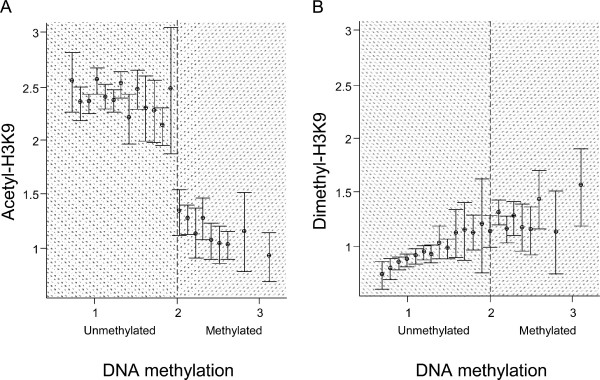
**The distribution of histone modifications against DNA methylation identified by ChIP-chip and DMH with CpG island array**. **A **and **B**. Positive targets (>2-fold change) from ChIP-chip (both acetyl-H3K9 and dimethyl-H3K9) and/or DMH are selected. The histone acetylation and methylation levels are plotted against DNA methylation status. Histone modification levels in L1210 cells were examined by comparison between ChIP DNA enriched with acetyl- and dimethyl-H3K9 antibody, and the input DNA. DNA methylation status was screened by DMH at *Bst*UI and *Hpa*II sites. Mean plots with error bounds were used to indicate the level of histone modifications.

Next, DMH was performed using the mouse CpG island microarray. Because L1210 originated from the mouse strain DBA2, we used genomic DNA derived from this mouse strain as a control for assessing DNA methylation in L1210. The DMH assay was used to evaluate the methylation status of *Bst*UI and *Hpa*II sites, located within or nearby CpG islands. A 2-fold increased intensity was used as a cutoff for scoring positive loci for DNA methylation [23]. Those loci scoring positive (>2-fold) in DMH and/or ChIP-chip data were then used to compare the acety-H3K9 and dimethyl-H3K9 levels against their promoter methylation level (Figure 2). Overall, we observed a trend that high levels of acetyl-H3K9 (>2-fold) were preferentially present in unmethylated CpG islands while acetylation levels less than 2-fold are correlated with hypermethylated loci in L1210 (Figure 2A). However, the distribution pattern of dimethyl-H3K9 against DNA methylation status was not as distinct as that of acetyl-H3K9 in this cell line (Figure 2B).

### Subpanel profiling of acety-H3K9, dimethyl-H3K9, and DNA methylation in L1210 cells

**Figure 3:**
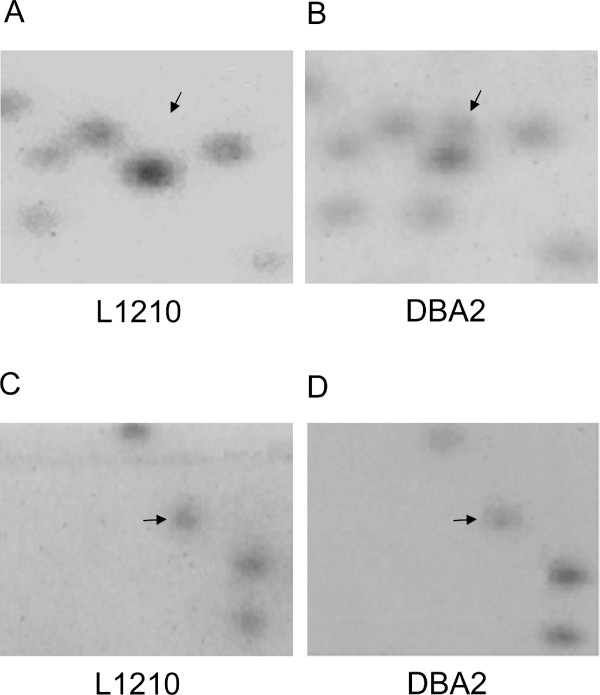
**RLGS analysis**. **A **and **B**. RLGS sections from L1210 and DBA2. Fragment (arrow) was present in DBA2 mouse but lost in L1210, indicating that the gene was methylated in L1210 and resistant to methylation-sensitive *Not*I restriction enzyme digestion. **C **and **D**. Sections showing RLGS fragment (arrow) detected in both L1210 and DBA2 mouse, indicating that the gene was cut by *Not*I and not methylated in both L1210 and DBA2.

To independently confirm these genome-wide findings, we focused the epigenetic analysis to a subset of promoter CpG islands. We first used the restriction landmark genome scanning (RLGS) technique to identify hypermethylated loci in L1210 compared to the control DBA2. Loss of CpG island sequences in RLGS indicates potential *Not*I hypermethylation present in this leukemia cell line (Figure 3) [24]. Of the 1300 sites screened, we identified a total of 435 (or 33%) RLGS fragment losses. The identified DNA methylation pattern of L1210 was similar to profiles obtained from the leukemia samples derived from a mouse model of NK/T acute lymphoblastic leukemia, with numerous commonly methylated sequences (data not shown) [25].

**Figure 4:**
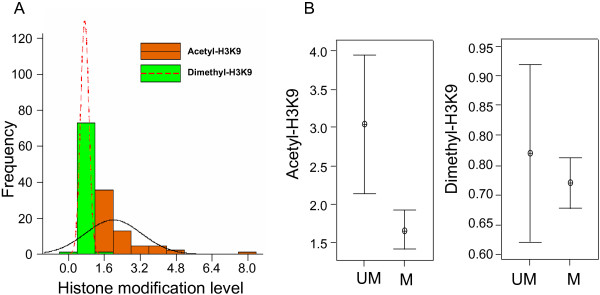
**Histone modifications and DNA methylation status in mouse promoter regions**. **A**. Histogram of histone acetylation and dimethylation identified by ChIP-chip with mouse custom promoter array. Brown and green bars indicate the frequency of acetyl-H3K9 and dimethyl-H3K9. Frequency curves are shown as follows: continuous line, acetyl-H3K9; dash line, dimethyl-H3K9. **B**. Interval plots of histone modifications versus DNA methylation. Histone modifications were plotted with two groups: unmethylated and methylated genes identified by RLGS. The interval bars indicate the distribution of histone modifications with horizontal lines at the endpoints of the 95% confidence interval for the mean and a symbol at the mean.

We then used a subset of 71 (including 54 hypermethylated loci and 17 unmethylated sequences) promoter CpG islands identified in RLGS to establish a custom mouse promoter array for ChIP-chip assays. The 5' fragments of these targets, including their transcription start site or *Not*I restriction site, were amplified by PCR and printed on glass slides (see Table S1 for location). Immunoprecipitated DNAs from L1210 using antibodies against acetyl- or dimethyl-H3K9 were then used to hybridize this custom microarray panel. The results showed that the level of acetyl-H3K9 had a wider range of distribution than that of dimethyl-H3K9 (Figure 4A). Similar to the results obtained from the global microarray, the dimethyl-H3K9 level of these 71 loci was not as high as expected even though most of the targets were methylated in L1210. To clarify the relationship between histone modifications and DNA methylation, we plotted the distribution patterns of histone acetylation or methylation *versus *DNA methylation in these 71 loci (Figure 4B). Consistent with the previous reports [26], this interval plot analysis showed that acety-H3K9 was reversely correlated with DNA methylation (*p *< 0.01). However, dimethyl-H3K9 and DNA were not found to be significantly correlated. Altogether, the results confirmed the aforementioned genome-wide findings that acetyl-H3K9 and dimethyl-H3K9 may have distinct autonomies with respect to DNA methylation in this subpanel of loci in L1210 cells.

### Confirmation of acety-H3K9, dimethyl-H3K9, and DNA methylation profiles in individual CpG island loci

**Figure 5:**
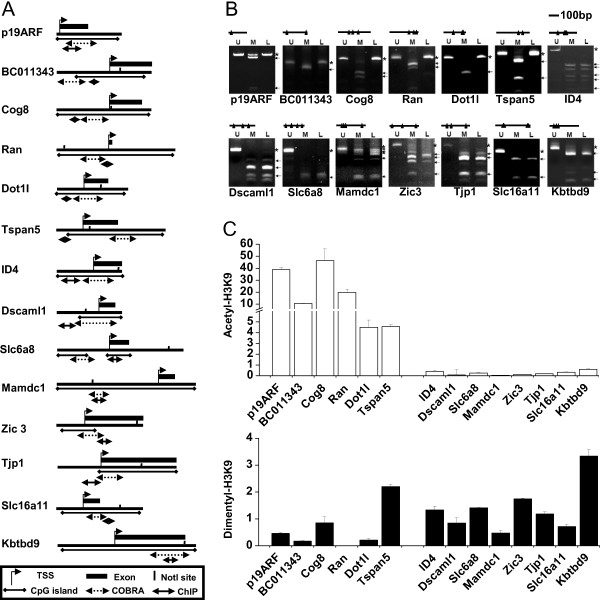
**DNA methylation and histone modifications identified by COBRA and ChIP-PCR**. **A**. The targets examined by COBRA and ChIP-PCR. *Not*I sites and regions for COBRA and ChIP-PCR are indicated. **B**. DNA methylation identified by COBRA. DNA from DBA2 mouse (U) and bisulfite treated DNA (M) were negative and positive controls, respectively. DNA from L1210 (L) was compared with controls. Arrows and stars indicate the methylated and unmethylated fragments, individually. **C**. Quantitative ChIP-PCR was conducted to examine the individual histone modifications of the same 12 selected genes including both methylated (right portion of graph) and unmethylated at CpG sites (left portion of graph). The histone modification level of each gene was compared with input genomic DNA from L1210 and indicated with bars: blank, acetyl-H3K9; black, dimethyl-H3K9. Each error bar represents the standard deviation calculated from triplicates.

To further confirm the DNA methylation status and histone modifications in individual genes, combined bisulfite restriction analysis (COBRA) and ChIP-PCR were performed on 12 genes chosen from the subpanel list (Table S2 and Figure 5A). Two known genes, *p19ARF *and *ID4*, were used as unmethylated and methylated controls, respectively (Figure 5)[25,27]. Of these selected genes, six were deemed active, while the rest were inactive, according to their associated epigenetic marks (Table S2). Of the 12 targets examined, the methylation status of 10 genes was confirmed in L1210 cells (Figure 5B). Two genes (*BC011343 *and *Dscaml1*), found to be methylated by RLGS (Table S1), were not confirmed by COBRA, which may be caused by different restriction enzymes used in these two methods. The COBRA data showed an "all or nothing" methylation status in the promoters of targets examined. In the tested sites of these promoters, there seems to be no partial methylation detected by COBRA, which facilitates our further analysis.

ChIP, followed by real-time PCR, was applied to evaluate both acetyl- and dimethyl-H3K9 enrichment in the promoter regions of these targets. The enrichment levels of these 12 loci were compared between ChIP-DNA and genomic DNA (Figure 5C). Eleven of the 12 loci showed the same trend of histone modifications as derived by ChIP-chip assays (Table S2 and Figure 5C). In this regard, the acetyl-H3K9 level was inversely correlated with the DNA methylation status. Not surprisingly, the dimethyl-H3K9 level showed no significant difference with respect to the DNA methylation status of these loci, suggesting that the acquisition of dimethyl-H3K9 is less dependent on DNA methylation in the protein-coding genes than acetyl-H3K9. Meanwhile, the overall enrichment of acetyl-H3K9 varied greatly, but dimethy-H3K9 varied to a lesser extent, which is consistent with our ChIP-chip data. The results of the two control genes, *p19ARF *and *ID4*, are consistent with our other data (Figure 5C).

**Figure 6:**
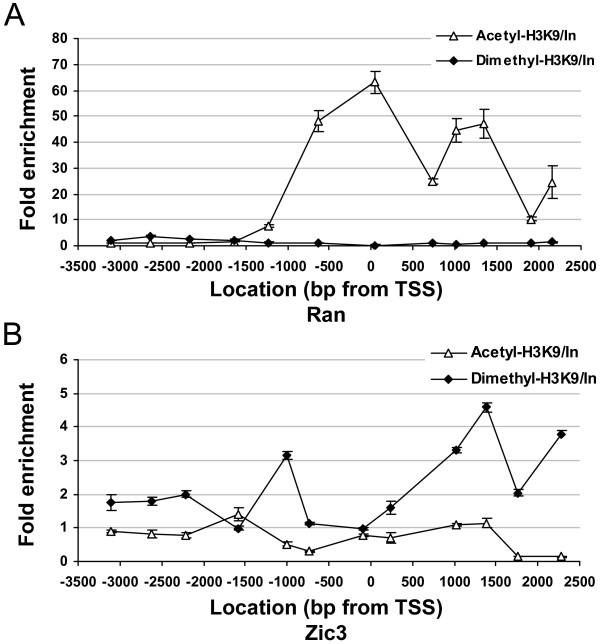
**The promoter landscape of histone modifications**. The fold enrichment by ChIP-chip with acetyl-H3K9 and dimethyl-H3K9 antibodies was screened by 12 sets of primers. The 12 PCR positions for each gene are indicated as the distances from transcription start site (0). Each error bar represents the standard deviation calculated from triplicates.

The chromatin landscape of the promoter regions (6-kb) in two genes, *Ran *and *Zic3*, was analyzed in greater detailed by ChIP-PCR. Twelve sets of primers were used in ChIP-PCR to cover their promoter regions from -3.5 to 2.5-kb away from the respective transcription start sites. The overall levels of acetyl-H3K9 were lower than those of dimethyl-H3K9 in the promoter region of the inactive *Zic3 *gene, but significantly increased in the active *Ran *gene in L1210 cells (Figure 6). In contrast, dimethyl-H3K9 levels were only slightly higher than those of acetyl-H3K9 in most parts of the interrogating regions, except in some regions of the active *Ran *promoter. The highest level of dimethyl-H3K9 was found in transcribed regions but not in the promoter of repressed genes.

### The effects of Trichostatin A (TSA) and 5-aza-2'-deoxycytidine (AzadC) on gene re-expression

**Figure 7:**
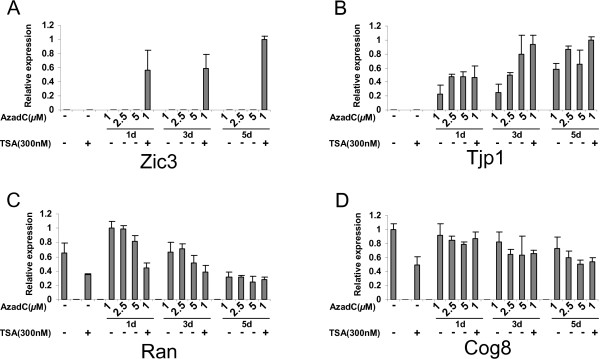
**The effect of TSA and AzadC on gene expression**. L1210 cells were treated without or with TSA (300 nM for 24 h), AzadC (1, 2.5 and 5 *μ*M for 1, 3 and 5 days), or the combination of TSA and AzadC (1 *μ*M of AzadC for 1, 3 and 5 days followed by 300 nM of TSA for 24 h). Expression of targets were identified by quantitative RT-PCR. **A **and **B**. *Zic3 *and *Tjp1 *are methylated and associated with hypoacetyl-H3K9. **C **and **D**. *Ran *and *Cog8 *are unmethylated and associated with hyperacetyl-H3K9. Each error bar represents the standard deviation calculated from triplicates.

Four genes were studied following treatment with TSA, a histone deacetylase inhibitor, and AzadC, a DNA methyltransferase inhibitor. The expression of four selected genes, *Tjp1*, *Zic3*, *Ran *and *Cog8*, were examined under different dosage of treatments for 1, 3 and 5 days. *Zic3 *and *Tjp1 *were associated with repressive epigenetic markers (Figure 5B and 5C), and their transcription in L1210 could not be detected with quantitative RT-PCR prior to drug treatment (Figure 7A and 7B). AzadC alone was able to de-repress *Tjp1*, but not *Zic3*. TSA alone did not reactivate *Tjp1 *or *Zic3*. However, a combination of TSA and AzadC was able to activate the expression of *Zic3*, and showed a synergetic effect. For *Tjp1*, the addition of TSA, however, had no or little additional effect on transcription, and its re-expression was due to AzadC treatment. Even though histone acetylation and DNA methylation were closely correlated for both *Tjp1 *and *Zic3*, these two epigenetic markers may affect gene function to differing extents. These results are also consistent with previous reports that DNA methylation is a dominant repressive factor, and that TSA alone may not de-repress gene transcription if the gene is densely methylated [15]. Two genes, *Ran *and *Cog8*, were expressed in L1210 and not methylated in their promoters (Figure 5B and 5C). The expression of *Ran *was increased under low concentrations (1–5 μM) of AzadC at day 1. However, both *Ran *and *Cog8 *showed reduced expression to varying degrees following prolonged treatments (5 days) of AzadC and/or TSA (Figure 7C and 7D).

## Discussion

The relationship between histone modifications and DNA methylation in maintaining gene silencing has been studied at the chromosomal level [28]. The model shows that a cooperation between methyl-H3K9 and DNA methylation is found in heterochromatin regions and major satellite repeats [28]. In euchromatic regions or at the individual gene level, controversial results have been reported in regards to the distribution and function of histone methylation in mammals [17,18,20-22].

Using genome-wide profiling techniques in L1210 leukemia cells, our present study shows distinct levels of autonomy in histone modifications in relation to DNA methylation of multiple protein-coding genes. Specifically, we demonstrate an inverse relationship between DNA methylation and histone acetylation in regulating transcription of these genes in mouse leukemia cells. However, methyl-H3K9 seems to be ambiguous in specifying silencing of some genes tested. Our findings might not fit into the model that histone methylation and DNA methylation are closely corollated in maintaining the repressive state of genes [3]. It should be noted that the establishment of this prior model is solely based on the observation of a few genes [11,12,14,29,30]. The present findings, however, are based on genome-wide profiling of these epigenetic components in multiple genes. Here we would like to propose an alternative model, in which histone methylation is distributed throughout the whole genome, including transcribed regions, and can be reversed by histone demethylase. However, DNA methylation is the final repressive lock, which can not be easily removed. In the "context" of stabilized chromatin, regions of histone acetylation "islands" are used to keep the active conformation at specific positions.

In addition to the above explanation, one additional suggestion is that the promoter region of protein-coding genes is not the prime target for this histone modification (i.e., methyl-H3K9). Recent discoveries have shown that the mouse promoter regions of *hemoglobin beta adult major chain *and *GATA-2 *have lower levels of both di- and tri-methylation of H3K9 than those in major satellite repeats and transcribed regions [31]. It is possible that regulatory mechanisms of histone methylation of H3K9, especially its crosstalk with DNA methylation, are different depending on chromatin locations and other unknown factors.

Our data shows the diverse status of histone modifications in relation to DNA methylation in mouse leukemia cells, providing new clues to the understanding of epigenetic regulation in mouse genome. In this regard, epigenetic components that specify active or inactive chromatin play different roles, but are cooperative, under different circumstances during mammalian development. Crosstalk between H3K9 methylation and DNA methylation are evolutionarily conserved from fungi to plants to mammals [3,32]. While genetic studies have shown that while H3K9 methylation is completely responsible for establishing DNA methylation in heterochromatic regions of *Neurospora*, this correlation is only partially established in *Arabidopsis *[6,7]. Meanwhile, some reports have shown that the distribution of histone methylation is dependent on DNA methylation in plants, but not in fungi [6,33]. In mouse embryonic stem cells lacking Dnmt1, Dnmt3a or Dnmt3b, no trimethyl-H3K9 redistribution is observed [8]. In double-null mouse ES cells for Suv39h, a histone methyltransferase, DNA methylation profiles are only changed in pericentric satellite repeats, but not in other repeat sequences [8]. Another histone methyltransferase, G9a, specifically affects imprinted genes depending on the development of embryonic stages [30]. Other gene studies have also produced controversial results between the correlation of histone modifications and DNA methylation [11,34,35]. From these studies, it is obvious that epigenetic redundancy, resulting from complex interactions among different chromatin components, is implemented to safeguard the stability of repressed chromatin structure.

Possible "heterogeneity" of epigenetic regulation is also revealed by our TSA/AzadC treatment study. Repressive epigenetic marks may be different in the 4 genes analyzed, as the same drug treatment produced differential effects of expression in these loci. Meanwhile, alternative pathways may exist for TSA or AzadC that affect their upstream regulators genes, which also regulate the expression of these genes.

Here we need to keep in mind that only dimethylation of H3K9 was examined in this study, and further investigation is essential to delineate the relation of mono- and trimethyl-H3K9 methylation to DNA methylation. It is known that in mouse, different types of methylation at H3K9 are distributed with various patterns in chromatin [3,32]. For example, trimethyl-H3K9 is over abundant in heterochromatin, whereas mono- and dimethyl-H3K9 are predominantly in euchromatin. Since we were more interested in the epigenetic modifications in euchromatin, dimethyl-H3K9 was selected in this study. Both mono- and trimethyl-H3K9 methylation deserve further study so that "heterogeneity" of epigenetic regulation can be well understood. We selected a single mouse leukemia cell line, L1210, in this study and conclusions drawn in this system will need to be validated in other mammalian cells.

ChIP-chip, RLGS, and DMH are genome-wide techniques, which can be readily applied to epigenomic studies. CpG island arrays have been widely used in human epigenetic studies [36]. However, very little work has been done combining mouse CpG island arrays with other epigenomic tools. Current knowledge of crosstalk between histone modifications and DNA methylation comes mainly from a series of experimental strategies, including complex interaction, genetic studies and sequence characterization [9]. In addition, one main direction is to delineate specific epigenetic marks that implicate cellular functions, such as cell-lineage determination and stem cell differentiation. These challenges require epigenomic tools, as those described in this study and two recent reviews [3,32]. Studies have described the use of ChIP-chip to investigate the correlation between histone modifications and gene transcription from yeast to human [37]. Because of technical limitations, few epigenomic tools have been reported in the mouse. The implementation of CpG island and custom microarrays makes it possible to interrogate complex epigenetic networks in mammalian systems.

## Conclusion

We have performed integrative epigenomic studies and found a diverse relationship between histone modifications and DNA methylation for the maintenance of gene silencing. Acetyl-H3K9 appears to have an inverse relationship with promoter methylation in protein-coding genes. In contrast, methyl-H3K9 seems to be less distinctly related to promoter methylation. This work also demonstrates the importance of using genome-wide approaches to decipher complex epigenetic regulation in the cell.

## Methods

### Cell culture

Mouse leukemia cell line L1210 (American Type Culture Collection, Manassas, VA) was grown in Dulbecco's modified Eagle's medium (Cellgro, Herndon, VA) plus 10% FBS in plastic tissue culture plates in a humidified atmosphere containing 5% CO_2 _at 37°C. The cells were grown to 90% confluency before being harvested.

### Chromatin immunoprecipitation microarray (ChIP-chip)

Five millions of L1210 cells were crosslinked with 1% formaldehyde for 10 min, and then 0.125 M glycine was used to stop the crosslinking. Chromatin immunoprecipitation was performed by using ChIP assay kit (Upstate Biotechnology, Charlottesville, VA) as described previously [38]. The antibodies against acetyl-H3K9 (AcH3K9, 06–942) and dimethyl-H3K9 (diMeH3K9, ab-7312) were purchased from Upstate Biotechnology (Charlottesville, VA) and Abcam (Cambridge, MA), respectively. Pooled DNA (up to 10) from multiple ChIPs and input DNA were labeled by Cy5 and Cy3 fluorescent dyes (Amersham, Buckinghamshire, UK) and then were cohybridized to the mouse 9.2k CpG island array (UHN microarray center, Ontario, Canada) or mouse custom array. Post-hybridization washes were performed as previously described [23]. The washed slides were scanned by a GenePix 4000A scanner (Axon, Union City, CA), and the acquired microarray images were analyzed with GenePix Pro 6.0 software (Axon, Union City, CA). Duplicate hybridizations were performed for each antibody and the quality of replicate chips was examined by scatter plot and Pearson's correlation analysis (from 0.77–0.82) [23]. After excluding the spots flagged for bad quality, normalized Cy5/Cy3 ratios of these loci were calculated by GenePix Pro 6.0 [38].

### Differential methylation hybridization

Differential methylation hybridization (DMH) was performed essentially as described ([23,38]). Briefly, 2 μg of genomic DNA were digested by *Mse*I to produce small fragments and then H-24/H-12 PCR linkers (5'-AGGCAACTGTGCTATCCGAGGGA T-3' and 5'-TAATCCCTCG-GA-3') were ligated to the digested DNA fragments. The DNA samples were further digested with two methylation-sensitive endonucleases, *Hpa*II and *Bst*UI, and amplified by PCR reaction using H-24 as a primer. After amplification, DNA from L1210 and DBA2 was labeled with Cy5 and Cy3 dye, individually. Hybridization and later analysis were performed as described above in ChIP-chip section.

### Mouse custom array

PCR was performed to amplify the promoter regions (-700 bp to +300 bp from the transcription start site) with mouse genomic DNA as template (see table S1). To ensure the reproducibility of each PCR and to prevent nonspecific amplification, PCR products (500-bp on average) were individually verified by 1.2% agarose gel electrophoresis. PCR products and the control repetitive DNA were then mixed with 50% dimethylsulfoxide and spotted in triplicate to GAPS II coated slides (Corning, Acton, MA) by Affymetrix/GMS 417 Arrayer (Affymetrix, Santa Clara, CA). Arrays were incubated in a desiccator overnight. Spotted DNA was rehydrated by holding slides over boiling water for 5 seconds and then placed on a hot plate for 2 seconds. UV (300 mJ) cross-linking was used to immobilize spotted DNA. Slides were then stored in a desiccator at ambient temperature.

### Restriction landmark genome scanning (RLGS)

High molecular weight DNA was extracted from L1210 cells and DBA2 mouse tissue. Subsequently, RLGS was performed as previously described [39]. Paired RLGS profiles, obtained from L1210 and DBA2, were overlaid and the difference between the two profiles was detected by visual inspection. Analysis was independently validated by at least one additional investigator. All selected targets for ChIP-chip were analyzed by comparing the RLGS profiles from L1210 and DBA2.

### Combined bisulfite restriction analysis (COBRA)

*In vitro *methylated DNA (representing 100% methylated DNA) and the DNA from a DBA2 mouse (representing 0% methylated DNA) were used as controls. Two micrograms of DNA from L1210 cells was treated with 3 M sodium bisulfite overnight and then amplified by PCR. Primers were designed to amplify both methylated and unmethylated alleles of sodium bisulfite-treated DNA. PCR products were purified by the gel extraction kit (Qiagen, Valencia, CA) and then digested by *Bst*UI (CG↓CG) restriction enzyme (NEB, Ipswich, MA). The digested fragments were separated on an 8% polyacrylamide gel. The primers are listed in Table S3.

### Chromatin immunoprecipitation-quantitative polymerase chain reaction

ChIP was conducted the same way as in ChIP-chip. DNA pool from ChIP and input control was first measured by spectrophotometer (NanoDrop, Wilmington, DE). Quantitative PCR with SYBR green-based detection (Applied Biosystems, Foster City, CA) was performed as described previously [40]. In brief, primers are designed according the promoter structure of selected genes (Figure 5A). Quantitative ChIP-PCR values were normalized against values from a standard curve (50 to 0.08 ng, R^2 ^> 0.99) constructed by input DNA with the same primer sets. The primers are listed in Table S3.

### Trichostatin A (TSA) and 5-aza-2'-deoxycytidine (AzadC) treatment

Cells were split the day before treatment and then treated with TSA (Sigma, St. Louis, MO), AzadC (Sigma, St. Louis, MO) or the combination of the two drugs. 1, 2.5 and 5 μM of AzadC in demethylsulfoxide was applied to cells every 24 h for 1, 3 or 5 days. 300 nM TSA in demethylsulfoxide was used to treat cells for 24 h. For combination treatment, 1 *μ*M of AzadC daily for 1, 3 or 5 days was followed with 300 nM TSA for 24 h. Cells treated with medium containing dimethylsulfoxide served as a control.

### Quantitative reverse transcription-polymerase chain reaction

Total RNA was extracted from drug treated and untreated cells. Two μg RNA was first treated with DNase I (Invitrogen, Carlsbad, CA) to remove potential DNA contamination and then was reverse transcribed with SuperScript II reverse transcriptase (Invitrogen, Carlsbad, CA). Quantitative RT-PCR was performed by using SYBR green (Applied Biosystems, Foster City, CA) as a marker for DNA amplification on a 7500 Real-Time PCR System apparatus (Applied Biosystems, Foster City, CA). The relative mRNA level of a given locus was calculated by relative quantitation of gene expression (Applied Biosystems, Foster City, CA) with GAPDH mRNA (based on amplification efficiency) as an internal control.

## Authors' contributions

JW contributed the design of the study, participated in all the experiments and analysis and drafted the manuscript. DP participated in the analysis of microarray data. SW and YW carried out RLGS experiments. JCL contributed to the construction of mouse custom array. LTS was involved in revising the manuscript. CP and TMH contributed to the conception and design of the study, coordinated the study, and helped with writing the manuscript. All authors read and approved the final manuscript.

## Supplementary Material

Additional file 1 **Table S1**. PDF file containing the targets on selected custom mouse array and control genes unmethylated discovered by RLGS in mouse leukemia. Click here for file

Additional file 2 **Table S2**. PDF file containing the targets selected for COBRA and ChIP-PCR. Click here for file

Additional file 3 **Table S3**. PDF file containing primers used in COBRA, ChIP-PCR, promoter landscaping analysis and RT-PCR. Click here for file
