# Supplementary material for: Diverse histone modifications on histone 3 lysine 9 and their relation to DNA methylation in specifying gene silencing
Source: BMC Genomics. 2007 May 24;8:131. doi: 10.1186/1471-2164-8-131 (PMC1888705; doi:10.1186/1471-2164-8-131)
Supplement: Additional file 1 — Table S1. PDF file containing the targets on selected custom mouse array and control genes unmethylated discovered by RLGS in mouse leukemia. [file 1471-2164-8-131-S1.pdf]

Table S1. The targets on selected custom mouse array and control genes unmethylated discovered by RLGS in mouse leukemia.

Spot on RLGS	GB Accession	Name	NotI site	Not I position[a]	CpG island	chip position[b]	mouse leukemia(8)
5F75	AF002701	Gfra2	5’ end	-114	Yes	-543, 87	8[c]
3F70	AF105268	Gpc6	5’ end	1062	Yes	-295, 254	8
3F56	U36760	Foxg1	5’ end	662	Yes	1353, 1900	7
6D12	AK080865	mRNA	5’ end	518	Yes	-494, 37	7
2H54	BE863773	EST	5' end	-294	Yes	-291, 162	7
4E24	AB041591	Slc16a11	5’ end	482	Yes	-54, 417	7
5F70	AF288666	Sema6a	5’ end	88	Yes	-316, 211	7
5F59	AB021964	Igsf4	5’ end	806	Yes	-406, 122	7
2F37	BC063061	Sox3	5’ end	1087	Yes	-250, 258	7
2G41	X75018	Id4	5’ end	210	Yes	-529, 4	7
3F09	AF334801	Pcdh10	Body	2960	Yes	-486, 45	6
2G81	AK047294	Cstad	Body	10072	No	-215, 85	6
3C06	No EST	-	-	chr1:15,464,778	Yes	Chr1:15,464,320-15,464,777	6
3E75	AJ010949	Cacna2d3	5’ end	-777	Yes	-310, -845	6
1F21	D70849	Zic3	5’ end	676	Yes	195, 526	6
2G50	AY371925	Mamdc1	5’ end	-1144	Yes	-499, 44	6
5G21	D14340	Tjp1	5’ end	525	Yes	-290, 251	6
4F59	BC014822	Ephb3	5’ end	-34	Yes	-257, 249	6
4G54	BC065168	Kbtbd9	5’ end	571	Yes	26, 517	5
1F30	AK089717	mRNA	Body	39864	Yes	39447, 39940	5
4D49	D83206	Vmp	5’ end	-47	Yes	-249, 247	5
5E02	AK041673	mRNA	5’ end	451	Yes	-784, -282	5
5D36	AF191211	Nr4a3	5’ end	684	Yes	-111, 429	5
3D03	AF263913	Fign	5’ end	-361	Yes	-558, -97	5
2F57	BC058660	Tgfbli4	5’ end	-59	Yes	10, 522	5
3F14	AK013491	mRNA	5’ end	416	Yes	-357, 151	4
4B19	AK086839	EST	-		Yes		4
5C23	AK053913	Slit2	5’ end	-1655	Yes	-586, -190	4
4E58	AK039431	mRNA	5’ end	123	Yes	-558, -90	4
4D27	M95599	Hoxa2	5' end	401	No	-240, 349	4
4G96	BE987673	mRNA	5’ end	-1	Yes	-610, -137	3
3G60	AK033940	4631416L12Rik	Body	1756	Yes	-404, 97	3
4D26	No EST	-	-	chr10:	Yes	chr10:18,243,737-18,244,237	3
4G84	AK076525	mRNA	5' end	205	Yes	1995, 2504	3
2G34	AK004677	Klhl13	5’ end	22	Yes	-387, 125	2
2G35	AK039308	mRNA	5’ end	139	Yes	-472, 16	2
2G48	L24118	Tnfaip2	5’ end	732	Yes	-252, 215	2
2F89	BE985330	EST	5' end	6	Yes	-318, 180	2
1G51	Y00051	Ncam1	5’ end	451	Yes	-348, 127	2
3G119	AK080973	mRNA	5’ end	-173	Yes	-355, 130	2
2F72	BC011343	mRNA	5’ end	131	Yes	-648, -127	1
2G09	AK087498	mRNA	5’ end	443	Yes	-288, 224	1
3C13	AK006521	mRNA	5’ end	182	Yes	-426, 116	1
1E27	S75970	mRNA	5' end	-1098	Yes	-238, 236	1
5G61	AF487346	Dscaml1	5’ end	-57	Yes	-297, 153	1
2D30	No EST	-	-	chr10:40,268,207	No	chr10:40,268,715-40,269,277	0
1F25	s75907	mTR2R1	5’ end	-1098	Yes	-314, 150	0
2F39	AK044806	mRNA	Body	1642	Yes	-310, 189	0
6D20	AK052809	mRNA	5' end	53	Yes	329, 875	0
3E11	BC042784	mRNA	3' end	48262	Yes	48032, 48524	0
4G73	BC085500	Irx3	body	1653	Yes	-604, 7	0
6C25	AK078614	Ptprk	5' end	270	Yes	-462, 37	0
2C51	AK015334	mRNA	5’ end	-208	Yes	-414, 82	0
1D26	bf456945	EST	5'end	4	yes	-317, 138	0
1F08	AK075900	Ran	5'end	-575	yes	-528, 12	0
2G93	AK081709	Ppra	5’ end	-700	yes	-321, 160	0
3D04	AB041610	Cog8	5’ end	21	yes	-896, -418	0
3D07	AF121344	Tspan5	5’ end	86	yes	-314, 192	0
3d63	BC016883	Golga5	5’ end	-35	yes	-317, 99	0
3E'54	BI689556	ETS	5' end	617	yes	-374, 162	0
3F61	BC030401	mRNA	5' end	236	yes	-175, 331	0
3G35	BC008101	Rasl11b	5' end	-277	yes	-507, 27	0
3G90	BC069933	Npepl1	5' end	226	yes	-754, -264	0
4B20	AK086839	mRNA	5' end	333	yes	-321, 169	0
4C14	AF179424	gata4	Body	4463	yes	-370, 126	0
4C18	AF459435	Slc6a8	5’ end	781	yes	-30, 437	0
4D15	AK002749	Ndufa6	5’ end	19	yes	-465, 84	0
4E'29	AK051764	mRNA	5' end	-1590	yes	-331, 165	0
4E'47	AF113751	Nup210	5’ end	79	yes	-436, 109	0
5G22	AK037877	mRNA	5' end	64	no	-294, 218	0
5G90	AK009348	mRNA	5' end	-222	yes	-715, -206	0
5H105	BC010216	Rara	5' end	236	yes	-687, -162	0
5H33	No EST	-	-	chr4:119,672,344	yes	chr4:119,672,010-119,672,488	0
6G41	AY196089	Dot1l	5' end	227	yes	-268, 263	0
6G67	BB578608	ETS	5' end	246	yes	-402, 133	0
7E'09	AK122293	Dnajc6	5, end	272	yes	-407, 111	0

[a, b] The numbers in this column mean the distance from transcription start site.

[c] The numbers indicate methylated samples identified by RLGS from a total of 8 test samples.
