# Supplementary material for: Diverse histone modifications on histone 3 lysine 9 and their relation to DNA methylation in specifying gene silencing
Source: BMC Genomics. 2007 May 24;8:131. doi: 10.1186/1471-2164-8-131 (PMC1888705; doi:10.1186/1471-2164-8-131)
Supplement: Additional file 2 — Table S2. PDF file containing the targets selected for COBRA and ChIP-PCR. [file 1471-2164-8-131-S2.pdf]

Table S2. The targets selected for COBRA and ChIP-PCR.

RLGS ID	GB Accession	Name	CpG island	mouse leukemia(8)	RLGS[b]	A/in[c]	M/in[d]	Function
2F72	BC011343	mRNA	Yes	1[a]	-	↑	↓	Unknown
3D04	AB041610	Cog8	Yes	0	+	↑	↓	Component of oligomeric golgi complex 8.
5G61	AF487346	Dscaml1	Yes	1	-	↑	↓	Down syndrome cell adhesion molecule-like 1. cell adhesion molecule that can mediate cation- independent homophilic binding activity.
1F08	AK075900	Ran	Yes	0	+	↑	↓	Member RAS oncogene family. GTP-binding protein involved in nucleocytoplasmic transport.
6G41	AY196089	Dot1l	Yes	0	+	↑	↓	Histone H3 methyltransferase DOT1.
3D07	AF121344	Tspan5	Yes	0	+	↑	↓	Tetraspanin 5.
4C18	AF459435	Slc6a8	Yes	0	+	↓	↑	Solute carrier family 6 (neurotransmitter transporter, creatine), member 8. Required for the uptake of creatine.
2G50	AY371925	Mamdc1	Yes	6	-	↓	↑	MAM domain containing 1. May involved in cell-cell interactions
1F21	D70849	Zic3	Yes	6	-	↓	↑	Zinc finger protein of the cerebellum 3. Probably functions as a transcription factor in the earliest stages of the left-right (lr) body axis formation
5G21	D14340	Tjp1	Yes	6	-	↓	↑	Tight junction protein 1. Homologous to the product of the 'discs large-1' tumor suppressor gene of Drosophila. may be involved in tight junction.
4E24	AB041591	Slc16a11	Yes	7	-	↓	↑	Solute carrier family 16 (monocarboxylic acid transporters), member 11.
4G54	BC065168	Kbtbd9	Yes	5	-	↓	↑	Kelch repeat and BTB (POZ) domain containing 9.

[a] The numbers indicate methylated samples identified by RLGS from a total of 8 test samples.

[b] "-", Spot lost in L1210 identified by RLGS; "+", spot detected in L1210 by RLGS.

[c, d] A/in, M/in indicate the level of acetyl-H3K9 and dimethyl-H3K9.
