# Supplementary material for: Diverse histone modifications on histone 3 lysine 9 and their relation to DNA methylation in specifying gene silencing
Source: BMC Genomics. 2007 May 24;8:131. doi: 10.1186/1471-2164-8-131 (PMC1888705; doi:10.1186/1471-2164-8-131)
Supplement: Additional file 3 — Table S3. PDF file containing primers used in COBRA, ChIP-PCR, promoter landscaping analysis and RT-PCR. [file 1471-2164-8-131-S3.pdf]

Table S3. The primers used in COBRA, ChIP-PCR, promoter landscaping analysis and RT-PCR.

Name	5' Primer	3' Primer	Annealing temperature,ºC
**COBRA**			
p19ARF	ggtttttggttattgtgaggattta	aacctttcctacctaatccaaaatt	56
BC011343	tggggagtttgtttagtgttagagt	ctcccttaaaaaaacaacaccttc	55
Cog8	gtatttgtagttttgttggaag	taaaaaaaataaaaaataaaaacctcctaa	55
Ran	taattgttgtttaggagggagttag	ccaaatcctatatccacaaacac	55
Dot1l	ggttttgtgatttttataaagaggg	acttaaacctcaactccaacttctc	55
Tspan5	tgtttttatgaatttagaggatgtt	aaacctaactaccccctacc	55
ID4	atttgagatattttttttaaaattt	aaccacccaaactataacc	50
Dscaml1	tttaggttggggttggagtagtat	ccacataccataaaaaaaccctatt	55
Slc6a8	atagttatttggggttgggt	taaacctaaaaaatctcatatatatc	55
Mamdc1	ttattatttgtaggagagggaggat	cctctacccaacttaaacatctctac	55
Zic3	taggttagttggtttttattggtg	aaatttatcaatcctatccc	55
Tjp1	ggttgggtatgtttagtggtt	aaaaattcaaataaaacaaaaatcc	55
Slc16a11	ttatttttgtgttggttagttgttt	acctaaaaataaaaatcctaaatac	55
Kbtbd9	ggtattttaagagttttaatttgatt	aaacaaaaaaactaacttccctctc	55
**ChIP-PCR**			
p19ARF	ttatagatggactcggagcaagg	ccccctagcagtagctgcg	58
BC011343	gaggacggtccaggcttta	gacccggatgtacagggtaa	58
Cog8	ccccacctcacctcacatag	agcatcggagagaaccttca	58
Ran	ttgttgctccgccctctc	gactggagctggaaagatgg	58
Dot1l	tggtctctctcgctttcctc	accgacgcacgcacttac	58
Tspan5	aagccaccctctttttcagg	cgagagagacgagggaacac	58
ID4	ggcgatccaccttagtcgaag	tttgtgagcgacaatcggc	58
Dscaml1	tttccccttacatggcagac	cagtctcgatcctgctcctc	58
Slc6a8	aaagagcgctgaaaacggta	atctggcgtgtccaagtctc	58
Mamdc1	agagcagatccgcacacc	tctaaactcccgccagtctc	58
Zic3	cgcgctcttgagtagaggag	ggaggagaagggaggagaaa	58
Tjp1	cacaggagtttggggttctc	ttacttgctaggcggtttcc	58
Slc16a11	ctatccctaggcctggttcc	gtggggaaacacctgtgaat	58
Kbtbd9	aggagggttgatggataggg	aggaacgagaggaggtggac	58
**Promoter lanscaping**			
Ran-1	tagaggtgactcggtcgttaagagta	tggaactggaattacagaatagttgtg	58

Ran-2	taaacagatgcttaaaacaatgcactagt	ccatattctgtagtccatttgaaaacac	58
Ran-3	ctaccttcaatcaagttgaccacagat	acgcctgctctttcatatgtcttat	58
Ran-4	tggaaggcataatggtgagagtg	gggcagtaccctagactgaacaaa	58
Ran-5	atgcccgcttgagtgtattctc	tgtcctggcaaccagcct	58
Ran-6	ttgctggtcaattgctgctc	tccaggcgttcagcatcc	58
Ran-7	gtccgctgcgtctccg	cctcgacgctaccttccaga	58
Ran-8	ccgctcgtcttccatacca	ccttggatgtagtagccatcgc	58
Ran-9	tggtgtcgtttgatcatatgctg	tgacccgggagctttcc	58
Ran-10	taaagacgaatgaatatccttgtgatctt	accccggttataaacacccc	58
Ran-11	ctaactggcatagagatctggtacga	aatagattttgccttcactttcctgt	58
Ran-12	tttagaatgtcctggaatggagattat	agttgtagttacttttggcagaaatgtc	58
Zic3-1	atgtaaaccccagtaagccaaagt	tcagacaggaccactcgaacc	58
Zic3-2	ccacaacagaattcgaaatggtc	taggagtgcagttgttcagtgagg	58
Zic3-3	aggaatgctagtccccaactacc	cagatacccggataagcgagg	58
Zic3-4	gagggcagaaccggaaaag	ccatctctctgtagcaaacaacaact	58
Zic3-5	acacgaaaagcacagtcactgtct	gacacataccagaaacaagcatagatg	58
Zic3-6	ctgttctatcacgggacaaggg	gagcctactaacggtaattcggag	58
Zic3-7	gcgctgccaatcattgtgt	aatcactcactcctcgcacataaa	58
Zic3-8	cgcgctcttgagtagaggag	ggaggagaagggaggagaaa	58
Zic3-9	cgagcagtcttcacgctcc	gttgtccacgtgccctgtg	58
Zic3-10	aagaagagctgcgaccgga	ccaatagcagacgtggttgttc	58
Zic3-11	cgccgagacctttcagtacc	ggaacttagaactcggcaaaagc	58
Zic3-12	actaccaggcttagcaaaaccg	aaccaagctgggtaggacaatg	58
**RT-PCR**			
Zic3	tgcgacaagtcctacacaca	ctatagcgggtggagtggaa	58
Tjp1	gcagccaaagaaggcttaga	ggaggtcaaggaggaaaagg	58
Cog8	agttctgcactgccttcctg	gtccaaggtttccgtgctta	58
Ran	tgtgtggcaacaaagtggat	ctggcaagccagaggaaag	58
GAPDH	cggtgtgaacggatttggc	tttgatgttagtggggtctcgc	58
